# Prevalence of elevated lipoprotein(a) in cardiac rehabilitation patients — results from a large-scale multicentre registry in Germany

**DOI:** 10.1007/s00392-024-02427-0

**Published:** 2024-04-15

**Authors:** Christoph Altmann, Nelu-Adrian Burlacu, Thomas Preuss, Annett Hlousek, Stephan Eddicks

**Affiliations:** 1 Department for Cardiology, MEDIAN Rehabilitation-Centre Bad Gottleuba, Königstr. 39, 01816 Bad Gottleuba-Berggießhübel, Germany; 2 Department for Cardiology, MEDIAN Rehabilitation-Centre Bernkastel-Kues, Bernkastel-Kues, Germany; 3 Department for Research & Innovation, MEDIAN Headquarters, Berlin, Germany

**Keywords:** Lipoprotein(a), Aortic valve stenosis, Myocardial infarction, Cardiovascular risk, Cardiovascular prevention

## Abstract

**Background:**

Lipoprotein(a) (Lp(a)) is an independent risk factor for myocardial infarction and aortic valve stenosis. European guidelines recommend assessing it at least once in a lifetime, particularly in premature atherosclerotic heart disease.

**Methods:**

A non-interventional registry was conducted at MEDIAN rehabilitation facilities in Germany to assess the frequency of Lp(a) testing in referring acute care hospitals and the prevalence of elevated Lp(a) levels in aortic valve stenosis or premature myocardial infarction. All consecutive patients referred after coronary intervention or aortic valve surgery were included in four cohorts: aortic valve intervention (cohort 1), current/previous myocardial infarction at < 60 years of age (cohorts 2a/2b), and myocardial infarction at ≥ 60 years of age (control).

**Results:**

The analysis included 3393 patient records (cohort 1, *n* = 1063; cohort 2a, *n* = 1351; cohort 2b, *n* = 381; control, *n* = 598). Lp(a) had been determined at the referring hospital in 0.19% (cohort 1), 4.96% (cohort 2a), 2.36% (cohort 2b), and 2.01% (control) of patients. Lp(a) levels were > 50 mg/dL or > 125 nmol/L in 28.79% (cohort 1), 29.90% (cohort 2a), and 36.48% (cohort 2b; *p* < 0.001) compared to 24.25% (control). Family history of premature cardiovascular disease was reported in 13.45% (cohort 1), 38.56% (cohort 2a), and 32.81% (cohort 2b) compared to 17.89% (control; *p* < 0.05 for each comparison).

**Conclusions:**

Lp(a) had been rarely assessed in acute management of aortic valve stenosis or premature myocardial infarction despite expanding scientific evidence and guideline recommendation. Given the above-average incidence of elevated Lp(a) levels, awareness for Lp(a) has to increase substantially to better identify and manage high-risk patients.

**Graphical Abstract:**

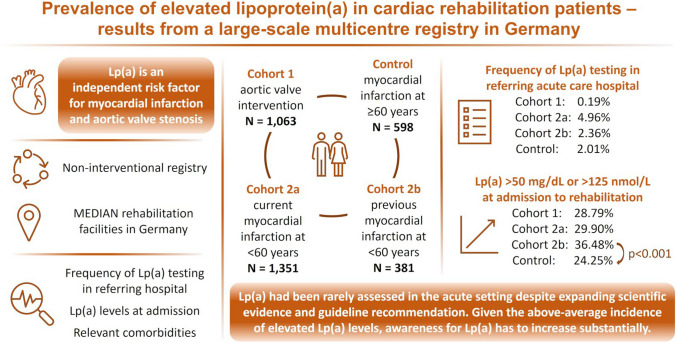

## Introduction

Myocardial infarctions in younger people are considered rare events but occur more frequently than generally assumed. It had been shown in 2015 that 1 in 15 myocardial infarctions affect a patient younger than 45 years; the majority of these are male [1]. These premature myocardial infarctions are often more severe with a higher 1-year mortality compared to older patients and lack a typical cardiovascular risk profile [2].

Knowledge about the role of lipoprotein(a) [Lp[a]) as an independent risk factor in the development of premature cardiovascular disease has expanded enormously in the past decade. Evidence has recently been worked up systematically in a consensus statement of the European Atherosclerosis Society [3]. The correlation between elevated levels of Lp(a) and the risk for myocardial infarction or aortic valve stenosis is statistically sound and has been established upon meta-analyses and Mendelian randomization study approaches [3]. For example, epidemiological data of the Copenhagen City Heart Study and the Copenhagen General Population study, together comprising over 77,000 participants, confirmed a significant increase in the risk of aortic valve stenosis and myocardial infarction already at Lp(a) levels > 30 mg/dL with a threefold risk at Lp(a) levels of > 90 mg/dL [4, 5]. The INTERHEART trial showed that Lp(a) levels > 50 mg/dL were associated with a significant increase in the risk of myocardial infarction [6].

Lp(a) levels are mainly genetically determined, remain almost constant for a lifetime, and cannot be impacted by lifestyle interventions [3]. Although strong evidence supports Lp(a) as an independent risk factor, it is not yet covered in current cardiovascular risk scores. However, the 2019 European ESC/EAS guidelines on the management of dyslipidaemia recommend that Lp(a) assessment should be considered at least once in lifetime to identify very high inherited Lp(a) levels [7]. Furthermore, international guidelines agree in the recommendation to determine Lp(a) as an additional risk marker in the case of premature atherosclerotic heart disease, with an upper limit of normal of 50 mg/dL or 125 nmol/L [7, 8]*.*

The recommendations of the ESC/EAS guidelines for comprehensive determination of Lp(a) have been implemented insufficiently in clinical practice in Germany. According to a claims data analysis, even in younger patients with atherosclerotic cardiovascular disease, Lp(a) testing is performed in less than 2% of cases [9]. Relevant data on the association between elevated Lp(a) levels and cardiovascular disease collected in clinical practice in Germany might help promote and raise awareness for Lp(a) as an important risk factor. The MEDIAN Lp(a) registry therefore aimed to determine the prevalence of relevant Lp(a) elevation in patients with premature myocardial infarction and aortic valve intervention referred for follow-up-treatment or rehabilitation and to analyse the frequency of Lp(a) assessments in the referring acute care hospitals.

## Methods

### Design and data collection

The present study was a non-interventional, multicentre registry conducted in MEDIAN cardiac rehabilitation facilities in Germany. Sites that did not have stable access to the electronic data registry were excluded from participating.

All consecutive patients who were referred to a cardiology department of the MEDIAN Group in Germany to receive follow-up treatment or rehabilitation after coronary intervention or aortic valve intervention were included. It was planned to include 3000 patient records into the registry within 1 year.

Four cohorts were assessed: patients with aortic valve intervention (cohort 1); patients after myocardial infarction at < 60 years of age (cohort 2a); patients with a history of myocardial infarction at < 60 years of age (cohort 2b); and patients with myocardial infarction at ≥ 60 years of age (control cohort; planned group size limited to 500 patients).

The following information were collected as part of the clinical routine: demographic parameters; prevalence of relevant comorbidities; lipid-lowering therapy status, anticoagulation status; relevant cardiovascular family history; smoking status including pack years; post hoc calculated cardiovascular risk score; prevalence of documented Lp(a) assessments from the referring hospital’s medical report; and laboratory parameters upon admission to rehabilitation including Lp(a) and low-density lipoprotein cholesterol (LDL-C). The data quality in the electronic data capture form was regularly checked by a central data management. Missing entries were queried subsequently. The main outcome was the prevalence of elevated Lp(a) levels defined as any Lp(a) level > 125 nmol/L or > 50 mg/dL upon admission to rehabilitation. Additionally, the prevalence of Lp(a) levels < 75 nmol/L or < 30 mg/dL upon admission to rehabilitation was analysed. Subgroup analyses on Lp(a) levels by gender and family history of premature cardiovascular disease were performed.

The registry was in accordance with all relevant guidelines and regulations applicable in Germany. According to local regulations, neither ethics committee approval nor registration was required due to the non-interventional design and the absence of any drug effectiveness assessment. Written informed consent to data collection, processing, and analysis was obtained from all patients prior to data collection. Patient data were anonymized and aggregated for analysis.

### Statistical methods

Only data sets which have been validated as complete have been included in the analysis. Plausibility checks have been performed on numerical vital and laboratory values in the database. Implausible values were excluded from analysis.

Categorical variables were summarized as number and percentages. Continuous variables were summarized as mean together with standard deviation (SD) as well as median together with minimum and maximum. No formal statistical hypothesis testing was applied. Statistical tests were applied to test for group differences with the control cohort as reference (chi-square test for categorical variables, two-sided Wilcoxon test for continuous variables; Holm’s method was used for pairwise comparison to adjust for multiplicity). Statistical analyses were performed using R, version 4.2.1.

## Results

Of 19 MEDIAN cardiac rehabilitation facilities in Germany, ten actively participated in the data collection. In total 3587 records were created from 01 March 2022 to 28 February 2023, of which 3393 records were validated as complete. Of these, 1063 patients belonged to cohort 1 (patients with aortic valve intervention), 1351 patients to cohort 2a (myocardial infarction at < 60 years of age), and 381 patients to cohort 2b (history of myocardial infarction at < 60 years of age). The control cohort included 598 patients (myocardial infarction at ≥ 60 years of age).

Patient demographics and clinical characteristics are shown in Table 1. Patients with aortic valve intervention in cohort 1 and patients with a myocardial infarction in the control cohort were approximately 70 years of age on average. In comparison, patients with premature myocardial infarction in cohort 2a and 2b were younger as per definition. Patients with myocardial infarction were predominantly male irrespective of the age of manifestation. Patients with aortic valve intervention in cohort 1 also included more male than female patients but with less pronounced difference in gender distribution compared to the other cohorts. The mean body mass index (BMI) ranged from 27.75 in the control cohort to 28.15 in cohort 1, 28.92 in cohort 2a, and 29.67 in cohort 2b with a statistically significant difference in cohorts 2a and 2b compared to the control cohort (*p* < 0.001 for both comparisons) (Table 1).

**Table 1:** Patient characteristics and comorbidities at admission

	Patients with aortic valve intervention Cohort 1 (*N* = 1063)	Patients with current MI at < 60 years Cohort 2a (*N* = 1351)	Patients with history of MI at < 60 years Cohort 2b (*N* = 381)	Patients with current MI at ≥ 60 years Control cohort (*N* = 598)
Age				
Mean (SD)	71.48 (11.13)***	52.18 (6.07)***	61.71 (8.35)***	69.89 (7.95)
Median (Min, Max)	72 (19, 94)	54 (27, 59)	61 (33, 84)	69 (60, 89)
Gender, *n* (%)				
Male	670 (63.03)**	1115 (82.53)***	319 (83.73)***	425 (71.07)
Female	392 (36.88)	236 (17.47)	62 (16.27)	173 (28.93)
Diverse	1 (0.09)	0 (0.0)	0 (0.0)	0 (0.0)
BMI				
* n* excluded^a^	1	3	1	3
Mean (SD)	28.15 (5.28)	28.92 (5.21)***	29.67 (5.57)***	27.75 (4.89)
Median (Min, Max)	27.6 (15.6, 54.8)	28.3 (14.4, 59.6)	28.8 (18.0, 53.5)	27.2 (17.6, 55.4)
Hypertension, *n* (%)	877 (82.50)	978 (72.39)	264 (69.29)*	464 (77.59)
Systolic blood pressure^b^				
* n* excluded^a^	0	0	1	0
Mean (SD)	134.59 (20.39)	131.48 (17.37)*	133.99 (20.08)	134.83 (20.69)
Median (Min, Max)	132 (90, 225)	130 (82, 216)	134 (60, 203)	134 (79, 196)
Diastolic blood pressure^b^				
Mean (SD)	76.62 (11.58)***	81.96 (11.15)***	80.26 (11.27)	79.28 (10.87)
Median (Min, Max)	76 (43, 130)	80 (28, 121)	80 (52, 115)	80 (48, 110)
Diabetes mellitus,* n* (%)	342 (32.17)	297 (21.98)***	136 (35.70)	199 (33.28)
Dyslipidaemia, *n* (%)	862 (81.09)***	1281 (94.82)	356 (93.44)	566 (94.65)
Lipid-lowering therapy, *n* (%)^c^				
Orals	862 (100.0)	1280 (99.92)	356 (100.0)	566 (100.0)
Injectables	1 (0.12)	4 (0.31)	3 (0.84)	2 (0.35)
Anticoagulation, *n* (%)	1042 (98.02)	1332 (98.59)	376 (98.69)	593 (99.16)
Family history premature CVD, *n* (%)	143 (13.45)*	521 (38.56)***	125 (32.81)***	107 (17.89)
Smokers				
Never, *n* (%)	698 (65.66)***	344 (25.46)***	100 (26.25)***	287 (47.99)
Former, *n* (%)	275 (25.87)**	537 (39.75)	187 (49.08)***	207 (34.62)
Yes, *n* (%)	90 (8.47)***	470 (34.79)***	94 (24.67)*	104 (17.39)
Pack years^d^				
* n* excluded^a^	0	3	0	0
Mean (SD)	23.88 (18.28)**	22.83 (16.02)***	28.66 (20.09)	28.42 (19.06)
Median (Min, Max)	20 (0.2, 120)	20 (0.3, 140)	25 (0.2, 135)	28 (0.2, 120)
ESC/EAS score				
* n*’	38	1351	150	598
Mean (SD)	6.01 (7.50)***	3.06 (2.48)***	4.96 (4.53)***	9.33 (7.19)
Median (Min, Max)	4.0 (0, 40)	2.8 (0, 25)	3.0 (0, 28)	7.3 (1, 49)

*BMI* body mass index, *CVD* cardiovascular disease, *MI* myocardial infarction, *Max* maximum, *Min* minimum, *N* number of patients in the cohort, *n* number of patients in the category, *n’* number of patients with data available, *SD* standard deviation

*p < 0.05; **p < 0.01; ***p < 0.001. Wilcoxon test (two-sided) for continuous variables; chi-square test for binary variables; Holm’s method was used for pairwise comparison to adjust for multiplicity. The control cohort served as test reference group

a: excluded values, BMI < 12 kg/m^2^ or > 59.7 kg/m^2^; systolic blood pressure > 230 mmHg; pack years > 200

b: among patients with hypertension

c: among patients with dyslipidaemia

d: among former or current smokers

Numerically fewer patients in cohorts 2a and 2b had hypertension compared to the other cohorts, with a statistically significant difference in cohort 2b compared to the control cohort (*p* < 0.05). The mean systolic blood pressure among patients with hypertension was 131 to 135 mmHg, and the mean diastolic blood pressure was 77 to 82 mmHg. The difference between cohort 2a and the control cohort was statistically significant (*p* < 0.05 for systolic blood pressure and *p* < 0.001 for diastolic blood pressure). However, the trend in the direction of the difference between cohorts was inconsistent; i.e. cohort 2a had marginally lower systolic blood pressure but marginally higher diastolic blood pressure compared to the control. The lowest prevalence of diabetes mellitus was reported in cohort 2a, and dyslipidaemia prevalence was the lowest in cohort 1. In both cases, the difference in prevalence rates was statistically significant compared to the control cohort (*p* < 0.001). All patients with dyslipidaemia except one in cohort 2a received oral lipid-lowering therapy at admission to the rehabilitation centre; less than 1% had already been escalated to injectable lipid-lowering therapy. Almost all patients received anticoagulation therapy (Table 1).

Family history of premature cardiovascular disease was reported in 38.56% of patients with current and 32.81% of patients with prior premature myocardial infarction. In contrast, family history of premature cardiovascular disease was reported in 13.45% of patients with aortic valve intervention. The differences in rates were statistically significant for all cohorts compared to the control cohort, in which 17.89% of patients had a family history of premature cardiovascular disease (*p* < 0.05 for cohort 1; *p* < 0.001 for cohorts 2a and 2b) (Table 1).

The proportions of former or current smokers were 25.87% and 8.47% in cohort 1 compared to 39.75% and 34.79% in cohort 2a as well as 49.08% and 24.67% in cohort 2b, respectively. Except for the proportion of former smokers in cohort 2b, all comparisons revealed a statistically significant difference versus the control cohort, in which 34.62% were former and 17.39% were current smokers (*p* < 0.05). Among former or current smokers, the difference in mean number of pack years was statistically significant in cohort 1 (23.88; *p* < 0.01) and in cohort 2a (22.83; *p* < 0.001) compared to the control cohort (28.42), but not for cohort 2b (28.66) (Table 1).

The highest median ESC cardiovascular risk score was 7.3 as reported in the control group with myocardial infarction at a higher age followed by 4.0 for patients with aortic valve intervention. The median ESC risk score was 2.8 in patients of cohort 2a with premature myocardial infarction and 3.0 for patients in cohort 2b with history of premature myocardial infarction. All comparisons showed a statistically significant difference versus the control cohort (*p* < 0.001 for all comparisons) (Table 1).

**Fig. 1:**
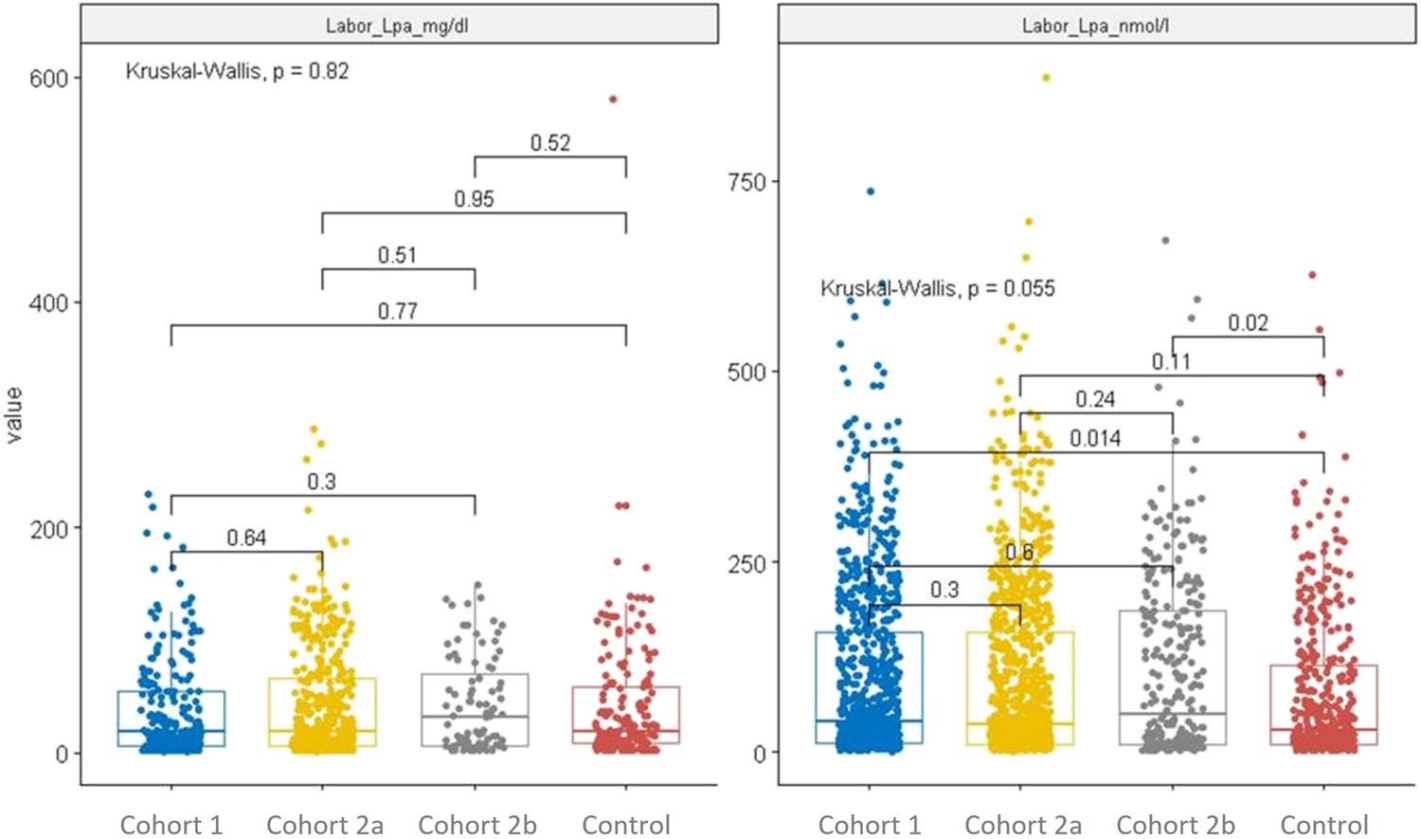
Lp(a) levels upon admission with interquartile range (box), the median value (line), and individual values (dots)

Lp(a) had been determined at the referring hospital in 2 patients (0.19%) of cohort 1, in 67 patients (4.96%) of cohort 2a, in 9 patients (2.36%) of cohort 2b, and in 12 patients (2.01%) of the control cohort. Lp(a) levels were assessed in all patients upon admission to rehabilitation (Table 2 and Fig. 1). Absolute Lp(a) levels (nmoL/L) in the combined cohorts were significantly different (*p* < 0.05) from the control group (Table 2). Elevated Lp(a) levels above 50 mg/dL or 125 nmol/L were detected in 28.79% of cohort 1, in 29.90% of cohort 2a, in 36.48% of cohort 2b, and in 30.38% in the combined cohorts, as well as in 24.25% in the control group (*p* < 0.001 for cohort 2b vs. control; *p* < 0.01 for the combined cohorts vs. control). In comparison, Lp(a) levels below 30 mg/dL or 75 nmol/L were detected in 61.43% of cohort 1, in 61.21% of cohort 2a, in 53.02% of cohort 2b, and in 60.18% in the combined cohorts, as well as in 65.22% in the control group (*p* < 0.01 for cohort 2b vs. control; *p* < 0.05 for the combined cohorts vs. control) (Table 2). Results of subgroup analyses of Lp(a) levels by gender and by family history of premature cardiovascular disease are shown in Tables 3 and 4.

**Table 2:** Laboratory parameters measured upon admission to the rehabilitation centre

	Patients with aortic valve intervention Cohort 1 (*N* = 1063)	Patients with current MI at < 60 years Cohort 2a (*N* = 1351)	Patients with history of MI at < 60 years Cohort 2b (*N* = 381)	Patients with current MI at ≥ 60 years Control cohort (*N* = 598)
Lp(a) in mg/dL				
* n*’	225	425	92	174
* n* excluded^a^	0	2	1	0
Mean (SD)	38.14 (45.03)	41.95 (48.69)	42.61 (41.69)	42.28 (61.05)
	40.87 (46.76)^b^			
Median (Min, Max)	18.40 (1.07, 230.00)	19.00 (1.40, 287.00)	31.90 (2.20, 149.00)	18.30 (2.20, 580.00)
	19.10 (1.07, 287.00)^b^			
Lp(a) in nmol/L				
* n*’	838	926	289	424
Mean (SD)	97.98 (117.93)	95.33 (118.17)	107.99 (120.34)	78.50 (100.51)
	98.19 (118.39)^b^*			
Median (Min, Max)	39.50 (1.00, 736.80)	37.05 (0.70, 886.00)	49.00 (1.80, 672.00)	28.70 (1.60, 626.00)
	39.00 (0.70; 886.00)^b^			
Elevated Lp(a), *n* (%)				
> 125 nmol/L or > 50 mg/dL	306 (28.79)	404 (29.90)	139 (36.48)***	145 (24.25)
	849 (30.38)^b^**			
< 75 nmol/L or > 30 mg/dL	653 (61.43)	827 (61.21)	202 (53.02)**	390 (65.22)
	1682 (60.18)^b^*			
LDL-C in mmol/L				
* n* excluded^a^	1	1	0	0
Mean (SD)	2.39 (0.98)***	2.10 (0.85)	2.22 (1.02)	2.08 (0.87)
Median (Min, Max)	2.17 (0.23, 6.77)	1.97 (0.05, 6.73)	2.05 (0.23, 7.28)	1.93 (0.39, 5.97)
LDL-C > 1.4 mmol/L, *n* (%)	916 (86.25)***	1078 (79.85)	309 (81.10)	470 (78.60)
Triglyceride in mg/dL				
* n* excluded^a^	3	1	0	0
Mean (SD)	132.48 (77.44)	147.71 (96.99)***	146.89 (78.98)***	134.19 (86.10)
Median (Min, Max)	114.04 (42.11, 1290.00)	128.95 (30.00, 2043.86)	129.00 (46.00, 835.09)	113.16 (37.00, 1197.37)
HbA1c in mmol/mol				
* n* excluded^a^	1	3	0	1
Mean (SD)	40.32 (8.58)***	42.27 (11.43)***	43.80 (10.87)	43.02 (9.58)
Median (Min, Max)	38.80 (21.31, 86.89)	38.80 (23.50, 131.69)	40.98 (19.13, 98.91)	39.89 (22.40, 96.72)

*HbA1c* haemoglobin 1c, *LDL-C* low-density lipoprotein cholesterol, *Lp(a)* lipoprotein(a), *MI* myocardial infarction, *Max* maximum, *Min* minimum, *N* number of patients in the cohort, *n* number of patients in the category, *n’* number of patients with data available, *SD* standard deviation

*p < 0.05; **p < 0.01; ***p < 0.001. Wilcoxon test (two-sided) for continuous variables; chi-square test for binary variables; Holm’s method was used for pairwise comparison to adjust for multiplicity. The control cohort served as test reference group

a: excluded values, Lp(a) > 1000 mg/dL; LDL-C > 30 mmol/L; triglyceride < 2 mg/dL; HbA1c < 10 mmol/mol or > 400 mmol/mol

b: combined cohorts 1, 2a, and 2b

**Table 3:** Lp(a) levels measured upon admission to the rehabilitation centre by gender

	Patients with aortic valve intervention Cohort 1 (*N* = 1063)	Patients with current MI at < 60 years Cohort 2a (*N* = 1351)	Patients with history of MI at < 60 years Cohort 2b (*N* = 381)	Patients with current MI at ≥ 60 years Control cohort (*N* = 598)
Lp(a) in mg/dL				
Male				
* n’*	147	347	77	118
Mean (SD)	35.84 (42.03)	44.28 (48.91)	42.89 (40.95)	35.18 (41.92)
Median (Min, Max)	18.3 (1.1, 218.4)	21.9 (1.4, 287.0)	32.4 (2.2, 149.0)	15.4 (2.2, 220.0)
Female				
* n’*	78	76	14	56
Mean (SD)	42.47 (50.21)	31.35 (46.50)	41.06 (47.18)	57.25 (87.46)
Median (Min, Max)	19.1 (1.5, 230.0)	10.8 (1.8, 261.0)	13.1 (2.2, 137.0)	28.7 (2.2, 580.0)
* p*-value male vs. female	ns	*	ns	ns
Lp(a) in nmol/L				
Male				
* n’*	523	767	241	307
Mean (SD)	100.85 (119.43)	93.16 (114.59)	105.85 (111.32)	76.64 (100.19)
Median (Min, Max)	44.2 (1.0, 736.8)	34.0 (2.2, 696.0)	56.0 (1.8, 569.9)	28.5 (1.6, 626.0)
Female				
n’	314	159	48	117
Mean (SD)	93.23 (115.62)	105.75 (134.01)	118.71 (159.11)	83.38 (101.63)
Median (Min, Max)	35.1 (1.8, 592.5)	43.2 (0.7, 886.0)	38.1 (3.0, 672.0)	34.0 (2.7, 492.4)
* p*-value male vs. female	ns	ns	ns	ns

*Lp(a)* lipoprotein(a), *MI* myocardial infarction, *Max* maximum, *Min* minimum, *N* number of patients in the cohort, *n* number of patients in the category, *n’* number of patients with data available, *ns* not significant, *SD* standard deviation

*p < 0.05; **p < 0.01; ***p < 0.001. Wilcoxon test (two-sided) for continuous variables. The control cohort served as test reference group unless otherwise indicated

**Table 4:** Lp(a) levels measured upon admission to the rehabilitation centre by family history of premature cardiovascular disease

	Patients with aortic valve intervention Cohort 1 (*N* = 1063)	Patients with current MI at < 60 years Cohort 2a (*N* = 1351)	Patients with history of MI at < 60 years Cohort 2b (*N* = 381)	Patients with current MI at ≥ 60 years Control cohort (*N* = 598)
Lp(a) in mg/dL				
Family history of premature cardiovascular disease, yes				
* n*’	33	231	56	50
Mean (SD)	25.47 (33.06)	41.41 (45.96)	39.95 (38.63)	36.88 (40.00)
Median (Min, Max)	15.6 (2.2, 138.0)	18.3 (1.4, 190.0)	32.2 (2.2, 137.0)	22.2 (2.2, 138.0)
Family history of premature cardiovascular disease, no				
n’	192	192	35	124
Mean (SD)	40.32 (46.50)	42.61 (51.89)	46.87 (46.44)	44.46 (67.73)
Median (Min, Max)	19.4 (1.1, 230.0)	19.7 (1.8, 287.0)	24.7 (2.2, 149.0)	17.0 (2.2, 580.0)
* p*-value yes vs. no	ns	ns	ns	ns
Lp(a) in nmol/L				
Family history of premature cardiovascular disease, yes				
* n*’	110	290	68	57
Mean (SD)	129.28 (139.19)	95.32 (115.43)	128.13 (123.50)	93.92 (124.48)
Median (Min, Max)	81.7 (3.7, 592.5)	36.9 (1.8, 648.0)	102.0 (2.4, 569.9)	24.3 (2.7, 554.6)
Family history of premature cardiovascular disease, no				
* n*’	728	636	221	367
Mean (SD)	93.25 (113.74)	95.33 (119.48)	101.79 (118.94)	76.11 (96.24)
Median (Min, Max)	37.0 (1.0, 736.8)	38.0 (0.7, 886.0)	39.2 (1.8, 672.0)	29.3 (1.6, 626.0)
* p*-value yes vs. no	**	ns	ns	ns

*Lp(a)* lipoprotein(a), *MI* myocardial infarction, *Max* maximum, *Min* minimum, *N* number of patients in the cohort, *n* number of patients in the category, *n’* number of patients with data available, *ns* not significant, *SD* standard deviation

*p < 0.05; **p < 0.01; ***p < 0.001. Wilcoxon test (two-sided) for continuous variables. The control cohort served as test reference group unless otherwise indicated

LDL-C values measured upon admission are presented in Table 2. Mean LDL-C levels ranged from 2.08 to 2.39 mmol/L. The proportion of patients above the LDL-C target of 1.4 mmol/L as recommended by the ESC/EAS guidelines for secondary prevention ranged from 78.60% (control cohort) to 86.25% (cohort 1; *p* < 0.001 vs. control) (Table 2). The mean triglyceride levels were below the level for moderate hypertriglyceridemia in all cohorts with marginally higher values in cohorts 2a and 2b and a statistically significant difference in these cohorts compared to the control cohort (both *p* < 0.001) (Table 2). The mean HbA1c levels ranged from 40.32 mmol/mol in cohort 1 to 42.27 mmol/mol in cohort 2a and 43.80 mmol/mol in cohort 2b. A statistically significant difference was observed in cohort 1 and cohort 2a compared to the control cohort (43.02 mmol/mol; both *p* < 0.001) (Table 2).

## Discussion

Data from the MEDIAN Lp(a) registry show pathologically elevated Lp(a) levels in one quarter to one third of patients with aortic valve intervention or myocardial infarction. Upon admission to rehabilitation, elevated levels of Lp(a) above 50 mg/dL or 125 nmol/L were detected in 28.79% of aortic valve intervention patients. In patients with premature myocardial infarction, 29.90 to 36.48% of patients had elevated Lp(a) levels, and in the control group of patients with myocardial infarction at a later age, the proportion was 24.25%.

According to the Copenhagen Heart Study, 20% of the general European population have Lp(a) levels above 50 mg/dL [10]. Also, in a cohort of 52,898 patients admitted to a clinic for cardiology in Germany, only 18.4% were reported to have Lp(a) levels above 50 mg/dL. This cardiologic cohort study was not limited to patients with major cardiac events [11].The prevalence of pathologically elevated Lp(a) observed in the MEDIAN registry therefore is higher than in the normal population and even higher than in a general cardiologic cohort. Furthermore, our results indicate that elevated Lp(a) levels are more frequent in aortic valve stenosis and myocardial infarctions, especially in patients with early events. This is consistent with the established risk correlations of Lp(a) elevations [3]. Therefore, more attention should be paid to the assessment of Lp(a) levels.

According to the ESC/EAS recommendations, Lp(a) should be determined at least once in a lifetime [7]. This is especially important as Lp(a) elevation is an independent risk factor, increasing the risk for major cardiovascular disease even in patients with low risk according to current scores, e.g. ESC/EAS SCORE. In the MEDIAN registry cohorts, the median cardiovascular risk scores in patients with aortic valve intervention and current or prior premature myocardial infarction were 4.0, 2.8, and 3.0, respectively. Thus, according to the risk scores, these patients did not have a high cardiovascular risk. The slightly higher cardiovascular risk score in cohort 1 can be solely explained by the higher age of these patients compared to cohorts 2a and 2b. Despite similar mean age in cohort 1 and the control cohort, the risk score in cohort 1 is lower compared to the control cohort, which emphasizes that the current risk scores do not adequately cover the actual cardiovascular risk.

Risk factors as diabetes mellitus and hypertension were less common in patients with premature myocardial infarction. However, with respect to diabetes, this only applies to cohort 2a. In cohort 2b, diabetes was more frequent than in cohort 2a, which may also be related to the higher age of the cohort. Cohort 2b included patients with a history of premature myocardial infarction, however, neither the time since the event had been documented nor the time since diagnosis of comorbidities. Therefore, it remains unclear whether diabetes mellitus was already prevalent at the time of the event in patients of cohort 2b. At the time of admission to the rehabilitation clinic, HbA1c levels were unremarkable on average. It can therefore be assumed that the blood glucose levels were well adjusted in all cohorts. Regarding patients in whom hypertension was documented, the current systolic and diastolic blood pressure on average indicated an adequate therapeutic management.

Dyslipidaemia was less frequent in cohort 1 of patients with aortic valve intervention and presumably plays a smaller role here than in other cohorts. All patients with documented dyslipidaemia received lipid-lowering therapy, but almost exclusively oral medication was mentioned. The LDL-C values measured upon admission to our institutions suggested insufficient LDL-C adjustment. The ESC/EAS-recommended LDL-C target level of 1.4 mmol/L for high-risk cardiac patients is rarely achieved [7]. It has to be pointed out that in our data, it remains unclear how long it has been since a lipid-lowering therapy has been initiated. Nevertheless, at least in the patients with history of myocardial infarction in cohort 2b, it could be assumed that dyslipidaemia had been diagnosed some time ago. If that is the case, lipid-lowering therapy had not been escalated adequately. According to modelling approaches and clinical investigation, available therapeutic options, including injectable escalation therapies, are sufficient to allow target achievement in almost all high-risk patients [12, 13]. Average triglyceride levels were within the normal range in all cohorts.

The ESC/EAS recommendation for a general Lp(a) test is controversial. Parhofer and Laufs argue against an extensive determination of Lp(a) levels as specific drug therapy for lowering Lp(a) is not yet available. However, they point out the importance of cascade screening [14]. Our registry data shows that more than one third of patients with premature myocardial infarction had a relevant family history of premature cardiovascular events compared to only one fifth of patients with aortic valve intervention and in the control cohort. This supports the importance of cascade screening [15]. However, an effective cascade screening would initially require the determination of Lp(a) in the index patients, i.e. those with a current cardiovascular event. The present MEDIAN Lp(a) registry data show that the assessment of Lp(a) is rarely implemented in clinical practice in Germany. Lp(a) had been determined in less than 5% of cases in the setting of acute hospital care, even in patients with premature myocardial infarctions. In patients with aortic valve stenosis, we were able to identify Lp(a) levels determined prior to rehabilitation referral in only 0.19% of cases.

The observed Lp(a) determination rate is insufficient given the relevance of Lp(a) levels especially if other risk factors do not sufficiently explain the occurrence of events. Patients who have experienced a life-threatening event need clarification of the causes with appropriate intervention and counselling on how to prevent future events. Unfortunately, no specific drug therapy is currently available on the market for the reduction of Lp(a) levels, standard drugs for the treatment of dyslipidaemia do not show sufficient effect on Lp(a) levels, and the risk of re-events may not be adequately reduced by LDL-C reduction alone [3]. From the point of view of cardiological rehabilitation, elevated Lp(a) and a cardiovascular risk constellation therefore result in an enormous need for advice for those affected. It is essential for the treating physicians to support their patients through systematic and adequate patient information and training, and to provide them with offers of cascade screening, and participation in scientific studies. Furthermore, participation in specialized self-help groups (e.g. https://lipidhilfe-lpa.de, which was founded in Dresden in 2019) could promote patients understanding of the disease. This may significantly increase motivation for strict normalization of manageable risk factors. Therefore, we advocate a systematic determination of Lp(a) in risk constellations, with the setting of cardiac rehabilitation offering advantages here. The MEDIAN registry data demonstrated the feasibility of Lp(a) assessments in the routine of cardiological rehabilitation.

The current data demonstrate the urgent need to increase awareness for Lp(a) assessments. This could be achieved through inclusion of Lp(a) in the common cardiovascular risk scores. In the EAS Consensus Statement, the working group quantified the increase in risk due to Lp(a) as a function of baseline risk score. A lifetime risk for major cardiovascular events of only 5% based on the current risk scores nearly triples at an Lp(a) of 150 mg/dl to reach 13.6% [3]. Inclusion of Lp(a) values in the risk scores also requires test standardization. A lack of test standardization in the past certainly contributed to the reluctance to assess Lp(a) levels [16]. Today, most commercially available assays are based on the same methodology using nmol/L as standard unit (with conversion to mg/dL, if necessary). The available assays provide comparable clinical utility in terms of cardiovascular risk assessment [17].

The present data of the MEDIAN Lp(a) registry provide valuable and representative insights on the current state of Lp(a) determination in Germany. The registry included a large-scale patient population which is representative for rehabilitation after aortic valve stenosis and premature myocardial infarction. The annual incidence of aortic valve stenosis interventions in Germany was reported to be approximately 30,000 in 2019 [18]. The annual incidence of acute myocardial infarction in patients younger than 60 years of age was reported to be approximately 50,000 in 2009 [19]. Over 1 year, the registry included over 1000 patients after aortic valve stenosis-associated intervention and almost 1500 patients younger than 60 years of age receiving follow-up treatment after acute myocardial infarction. Therefore, the registry covers approximately 3% of annual incident cases with aortic valve stenosis interventions and premature myocardial infarction in Germany. Furthermore, the German healthcare structure is fully reflected in the participating MEDIAN facilities, which means that members from both statutory and private health insurances are referred. Referrers are clinics with different care mandates, i.e. from basic and standard care facilities to major regional and maximum care facilities.

Despite the favourable scale of the registry and the representative structural conditions at the participating sites, the present data bears some limitation. In this context, it is particularly important to mention that LDL-C levels are not known prior to referral for rehabilitation or at the time of the event. Furthermore, it was not known which lipid-lowering therapy the patients were receiving at the time of admission to the rehabilitation facility and how long they had been receiving lipid-lowering therapy. Therefore, the influence of LDL-C as a major risk factor for cardiovascular events cannot be comprehensively assessed in the cohorts. The same applies to further relevant comorbidities as diabetes mellitus or hypertension. Regarding previous Lp(a) assessments, it should be mentioned that apart from Lp(a) levels obtained from referral documents, no information on previous assessments was available and was not actively queried. It is therefore possible that Lp(a) had already been assessed prophylactically in some patients and that a determination in the acute setting may therefore have been obsolete.

Overall, the MEDIAN Lp(a) registry shows that Lp(a) assessment in the context of severe cardiac events is performed infrequently, and at the same time, it demonstrates the above-average incidence of pathological elevation of Lp(a) levels in patients with aortic valve stenosis or premature myocardial infarction. Given the established association of Lp(a) elevation and cardiac risk, significantly more attention needs to be paid to Lp(a) levels to better identify and manage patients at risk. The rehabilitation phase is an appropriate context for this.

## Data Availability

Data are available upon request from the corresponding author.
